# Potential of MRNA vaccines for mpox prevention: current evidence and future directions

**DOI:** 10.1097/MS9.0000000000003558

**Published:** 2025-07-10

**Authors:** Toufik Abdul-Rahman, Ogungbemi Evelyn Faith, Oyinbolaji Akinwande Ajetunmobi, Hala Ibrahim Thaalibi, Chinelo Geraldine Ikele, Gargi Gautam, Faith Olanrewaju Omotayo, Poulami Roy, Alhazan Amir Adebayo, Muslimat Abolore Mojeed, Tolulope Taiwo Kareem, Halima Ibrahim Ali, Rebecca Grace Atieno, Esther Oluwafeyisayo Ademeta, Neil Garg, Patrick Ashinze, Godfred Yawson Scott

**Affiliations:** a Department of Research, Toufik’s World Organization, Sumy, Ukraine; b Department of Public and International Affairs, University of Lagos, Akoka, Nigeria; c Faculty of Medicine, Beirut Arab University, Beirut, Lebanon; d Department of Veterinary Medicine, University of Nigeria, Nsukka, Enugu State, Nigeria; e Department of Medicine, Georgian National University SEU, Tbilisi, Georgia; f Department of Pharmacy, Ekiti State University Teaching Hospital, Ado Ekiti, Nigeria; g Department of Medicine, North Bengal Medical College and Hospital, Siliguri, India; h Department of Electrical and Biomedical Engineering, Abiola Ajimobi Technical University, Ibadan, Nigeria; i Physiotherapy Department, University of Lagos, Lagos, Nigeria; j Microbiology, Abubakar Tafawa Balewa University, Bauchi State, Bauchi, Nigeria; k Nursing Department, Comboni College of Science and Technology, Khartoum, Sudan; l Department of Pediatrics, Maseno University School of Medicine, Kisumu, Kenya; m Medicine and Surgery, University of Novi Sad, Novi Sad, Serbia; n Rowan-Virtua School of Osteopathic Medicine, One Medical Center Drive, Stratford, New Jersey; o Faculty of Clinical Sciences, University of Ilorin, Ilorin, Nigeria; p Department of Medical Diagnostics, Kwame Nkrumah University of Science and Technology, Kumasi, Ghana

**Keywords:** COVID-19, monkeypox, mRNA vaccines

## Abstract

In 2022, the presumption of monkeypox (mpox) to be of limited epidemiology shifted when a global outbreak was announced. Being a member of the Orthopoxvirus genus in the Poxviridae family, it’d been reported in over 82 countries with over 17 000 confirmed cases by July 2022, thus showing its capability for spreading rapidly. As the smallpox vaccine offers 85% cross-immunity against mpox, the outbreak highlighted the attenuation of global immunity against orthopoxviruses after the cessation of vaccination campaigns against smallpox. The mortality of this virus is higher in vulnerable populations such as children, pregnant women, the elderly, and immunosuppressed individuals. With treatment methods being limited to off-label use of antivirals, the need for urgent and efficient preventative measures is emphasized. At present, JYNNEOS (Modified Vaccinia Ankara-Bavarian Nordic), showing favorable safety, and ACAM2000, a live attenuated virus with a high risk of side effects, are two vaccines that are indicated for mpox immunization. However, neither of them has proven full safety, efficacy, and widespread accessibility against mpox. Hence, the use of mRNA vaccines has emerged as a better alternative to traditional vaccinations, as they leverage synthetic messenger RNA to instruct host cells to produce antigens, eliciting both humoral and cellular immune responses. Though they provided rapid scalability, adaptability to emerging viral variants, and an established safety profile after the COVID-19 pandemic, their usage in preventing mpox remains an area of research. This paper elucidates the potential of mRNA technology to address the unmet needs in mpox prevention. It also highlights the need for genomic surveillance, immunological insights, and innovative delivery systems.

**HIGHLIGHTS**

- Monkeypox (mpox) infection, an infection caused by the mpox virus, has emerged as a significant global health concern.
- Currently, the JYNNEOS and ACAM2000 are authorized traditional vaccines for mpox prevention. However, there are concerns regarding the partial efficacy of these vaccines in eliciting robust neutralizing antibody responses against mpox, emphasizing the need for next-generation vaccine platforms.
- This study elucidates the potential of mRNA technology to address the unmet needs in mpox prevention.

## Introduction

The monkeypox virus (MPXV), a member of the Orthopoxvirus genus in the Poxviridae family, has emerged as a significant global health concern. Characterized by double-stranded DNA, monkeypox (mpox) is closely related to the variola virus, the causative agent of smallpox^[1,2]^. Initially identified in 1958 in research monkeys at the State Serum Institute of Copenhagen, the virus garnered its name from this early discovery[3]. However, its first human infection was documented in the Democratic Republic of Congo in 1970, marking the beginning of a series of sporadic outbreaks primarily confined to Central and West Africa[4]. These endemic occurrences highlighted the zoonotic potential of mpox, as the virus is frequently transmitted from wild animals, such as rodents, to humans. The global epidemiological landscape shifted dramatically in May 2022 when the World Health Organization (WHO) declared a public health emergency due to an unprecedented outbreak of mpox across multiple non-endemic regions[5]. By July 2022, the virus had been reported in over 82 countries, with over 17 000 confirmed cases spanning all 6 WHO regions, underscoring its capacity for rapid, widespread dissemination[6].

Unlike previous outbreaks largely confined to endemic zones, the 2022 mpox outbreak displayed distinct epidemiological trends. Most reported cases occurred among men who have sex with men, with a significant proportion of infections associated with close physical or sexual contact[6]. This transmission dynamic marked a departure from historical patterns, wherein mpox was predominantly linked to zoonotic spillovers or household transmission. Furthermore, the outbreak underscored critical gaps in global immunity to orthopoxviruses, as smallpox vaccination campaigns ceased following the WHO’s declaration of smallpox eradication in 1980[7]. The waning of cross-protective immunity, once conferred by the smallpox vaccine, has left populations vulnerable to mpox, particularly in immunologically naïve cohorts.

The clinical manifestation of mpox varies, ranging from mild, self-limiting symptoms to severe systemic complications[8]. While the disease generally exhibits a low case fatality rate (3%–10%) in immunocompetent individuals, higher morbidity and mortality are observed in vulnerable populations, including children, pregnant women, the elderly, and individuals with immunosuppressive conditions such as HIV[9]. Current therapeutic options remain limited, relying primarily on off-label use of antivirals, including tecovirimat, cidofovir, and brincidofovir, which were originally developed for smallpox[10]. Despite these interventions, the absence of mpox-specific antiviral agents or FDA-approved treatments highlights the urgent need for targeted therapies and preventative measures.

Vaccination remains the cornerstone of mpox prevention. Historically, the smallpox vaccine demonstrated approximately 85% efficacy in preventing mpox infection[11]. However, the cessation of routine smallpox immunization programs has resulted in declining global immunity. Currently, two vaccines – JYNNEOS (Modified Vaccinia Ankara-Bavarian Nordic [MVA-BN]) and ACAM2000 – are authorized for mpox prevention[12]. JYNNEOS, a third-generation vaccine, has shown a favorable safety profile in clinical trials, making it the preferred option in non-endemic regions. Conversely, ACAM2000, a live attenuated vaccine, carries a higher risk of adverse effects, particularly in immunocompromised individuals. Despite these advancements, neither vaccine fully meets the global demand for safe, effective, and accessible mpox immunization. Recent studies have also raised concerns about the partial efficacy of these vaccines in eliciting robust neutralizing antibody responses against mpox, emphasizing the need for next-generation vaccine platforms[13].

mRNA vaccine technology has revolutionized the landscape of infectious disease prevention, offering a promising solution to the challenges posed by mpox. Unlike traditional vaccines, mRNA vaccines leverage synthetic messenger RNA to instruct host cells to produce antigens, eliciting both humoral and cellular immune responses. This platform’s unique advantages include rapid scalability, adaptability to emerging viral variants, and an established safety profile, as demonstrated during the COVID-19 pandemic[14]. The Pfizer-BioNTech and Moderna mRNA vaccines have been pivotal in curbing COVID-19, showcasing superior immunogenicity and efficacy compared to conventional vaccine modalities[15]. Moreover, the utilization of lipid nanoparticles (LNPs) as a delivery system has enhanced the stability and bioavailability of mRNA vaccines, enabling their widespread deployment in diverse populations.

Given the demonstrated success of mRNA vaccines against SARS-CoV-2, their application to mpox represents a critical avenue for research and development. Preclinical studies have already highlighted the potential of mRNA-based platforms in generating neutralizing antibodies and T-cell responses against orthopoxviruses[16]. However, comprehensive clinical evaluation is necessary to establish their efficacy, safety, and long-term immunogenicity in diverse demographic and immunological settings. Additionally, the cost-effectiveness and logistical feasibility of mRNA vaccine distribution must be addressed to ensure equitable access in low- and middle-income countries (LMICs), where the burden of mpox is disproportionately high.

This study aims to elucidate the potential of mRNA technology to address the unmet needs in mpox prevention. Furthermore, the study explores future directions for vaccine development, emphasizing the integration of genomic surveillance, immunological insights, and innovative delivery systems to enhance vaccine efficacy and global accessibility. This comprehensive review will contribute to the scientific discourse on mpox prevention, informing public health strategies and guiding the development of next-generation vaccines. The manuscript is in compliance with the Transparency in The Reporting of Artificial Intelligence Guidelines 2025[17].

## Traditional smallpox vaccines: historical perspective and mechanism

The history of smallpox vaccination represents a significant moment of immunological advancement, illustrating the relationship between scientific innovation and public health implementation. Smallpox, caused by the variola virus, was one of the most devastating infectious diseases known to humanity, with historical records suggesting its presence as early as the third century BCE[18]. The global impact of smallpox was profound, contributing to significant mortality, societal disruption, and economic burden. The eventual eradication of smallpox in 1980, facilitated by widespread vaccination, stands as one of the most significant achievements in the history of medicine[19].

The traditional smallpox vaccine, derived from the vaccinia virus, functioned through mechanisms that provided cross-protection against other orthopoxviruses, including variola, and to some extent, the MPXV[20]. Studies indicate that prior smallpox vaccination reduces the risk of mpox infection and attenuates disease severity. This cross-protection is attributed to the antigenic similarities among orthopoxviruses, which allow vaccine-induced immune responses to confer partial immunity against related pathogens. However, with the cessation of routine smallpox vaccination following eradication, population-wide immunity to mpox has waned, contributing to the increasing incidence of human mpox outbreaks in recent decades.

Understanding the historical development, immunological mechanisms, and efficacy of smallpox vaccination provides valuable insights into current and future vaccine strategies against poxviruses, including the development of novel mRNA vaccines for mpox prevention. The concept of immunization against smallpox predates modern vaccinology. Variolation, an early method of inducing immunity, was practiced in China, India, and the Ottoman Empire as early as the 10th century^[21,22]^. This technique involved introducing material from smallpox lesions into the skin or nasal passages of uninfected individuals, inducing a milder form of the disease that conferred immunity. While variolation significantly reduced mortality compared to naturally acquired smallpox infection, it carried inherent risks, including the potential for severe disease and onward transmission[21].

The shift in smallpox prevention occurred in 1796 with the pioneering work of Edward Jenner, a British physician who observed that milkmaids exposed to cowpox – a related orthopoxvirus – were resistant to smallpox[23]. Jenner hypothesized that exposure to cowpox could confer protective immunity against smallpox and tested this by inoculating an 8-year-old boy, James Phipps, with material from cowpox lesions[24]. Upon subsequent exposure to variola virus, Phipps did not develop smallpox, thus demonstrating the principle of vaccination. This discovery laid the foundation for the modern field of immunology and led to the widespread adoption of cowpox-derived vaccination as a safer alternative to variolation.

Throughout the 19th and early 20th centuries, advancements in vaccine production improved the safety and efficacy of smallpox immunization. Initially, vaccination was performed through direct arm-to-arm transfer, whereby material from a vaccinated individual’s pustule was used to inoculate others[25]. However, this method posed risks of transmitting other infectious agents, prompting the development of controlled propagation techniques. By the mid-20th century, the smallpox vaccine was produced in specialized facilities using calf lymph, ensuring standardized potency and purity[26]. The introduction of freeze-dried formulations further enhanced vaccine stability, facilitating mass immunization efforts, particularly in resource-limited settings[27]. The efficacy of the traditional smallpox vaccine stems from its ability to elicit robust and long-lasting immune responses. The vaccinia virus, an attenuated orthopoxvirus, serves as the immunogenic agent in the vaccine[28]. Upon inoculation, the virus undergoes limited replication at the site of administration, triggering a cascade of immune responses that protect against variola virus infection.

The immune response to vaccinia virus involves both humoral and cellular immunity. The primary humoral response is characterized by the production of neutralizing antibodies directed against viral surface proteins, including A27L, B5R, and D8L, which facilitate viral entry and dissemination[29]. These antibodies play a crucial role in conferring sterilizing immunity by preventing viral attachment and entry into host cells. Studies have demonstrated that vaccine-induced antibody titers remain detectable for several decades post-vaccination, contributing to long-term immunity[30].

In addition to humoral responses, cellular immunity is essential for effective viral clearance and long-term protection^[31,32]^. The vaccinia virus elicits strong CD8+ cytotoxic T lymphocyte (CTL) responses, which are critical for recognizing and eliminating infected cells. Memory T cells, generated following vaccination, provide durable immunity by rapidly responding to subsequent orthopoxvirus exposure. The role of CD4+ T helper cells is equally significant as they facilitate B cell maturation and enhance CTL responses. Collectively, these immune mechanisms establish a comprehensive defense against smallpox and related poxviruses.

The historical success of smallpox vaccination is evident in its role in the global eradication of smallpox. Before widespread vaccination programs were implemented, smallpox caused millions of deaths annually. The WHO launched the Intensified Smallpox Eradication Programme in 1967, employing mass vaccination campaigns and targeted ring vaccination strategies[33]. These efforts led to the interruption of variola virus transmission, with the last naturally occurring case reported in Somalia in 1977[34].

While highly effective, traditional smallpox vaccination is not without risks. The vaccine is a live virus preparation, which can cause localized reactions, including vesicular lesions at the inoculation site. In immunocompromised individuals, such as those with HIV/AIDS or underlying immunodeficiencies, vaccinia virus replication can lead to severe complications, including progressive vaccinia, eczema vaccinatum, and postvaccinal encephalitis[35]. These adverse events necessitate caution in vaccine administration, particularly in individuals with contraindications.

Additionally, the waning immunity in the post-eradication era raises concerns regarding the longevity of vaccine-induced protection[36]. Although antibody responses persist for decades, cell-mediated immunity declines over time, necessitating booster vaccinations for sustained protection. The increasing emergence of zoonotic poxviruses, including mpox, has renewed interest in developing safer and more targeted vaccines to address contemporary public health challenges.

Traditional smallpox vaccines’ historical development and immunological mechanisms prove their pivotal role in infectious disease control. By bringing about robust humoral and cellular immune responses, these vaccines not only facilitated the eradication of smallpox but also provided cross-protection against other orthopoxviruses. However, the cessation of routine vaccination has led to a decline in population-wide immunity, necessitating renewed efforts to address emerging threats such as mpox. The lessons learned from smallpox vaccination continue to inform the development of next-generation vaccines, including mRNA-based approaches, which hold promise for enhancing safety, efficacy, and scalability in the fight against poxvirus infections. As global health priorities evolve, leveraging historical successes while incorporating modern technological advancements will be critical in shaping future vaccination strategies against orthopoxvirus-related diseases.

## mRNAs and mRNA vaccines

### Role of mRNAs in the pathogenesis of viruses

mRNAs are a type of single-stranded RNA that plays a crucial role in the pathogenesis of viruses. They exist naturally as part of the genomes of some viruses (positive-sense RNA viruses) or may be produced by viral transcription in other viruses (negative-sense RNA and DNA viruses) following their entry into host cells. After host cell invasion, viral mRNAs are transcribed into viral proteins (enzymes and structural proteins or antigens) required for the replication and spread of the virus[37].

### Mechanism of mRNA vaccines

Traditional viral vaccines are developed with either weakened or dead viruses or their parts, and mainly work by triggering an immune response upon introduction into the body. While traditional vaccines have been used for decades, advances in mRNA vaccines have revolutionized the field of vaccinology due to their great application prospects and advantages, which include rapid development and production, flexibility to respond to new variants, and capacity to induce a better immune response^[38–42]^.

Unlike traditional vaccines, mRNA vaccines consist of synthetic mRNA molecules that encode specific viral antigens, enabling host cells to generate these viral proteins and trigger immune responses. Like endogenous mRNA, they contain five structural elements: a 5ʹ cap, a 5ʹ untranslated region, an open reading frame, a 3ʹ untranslated region, and a poly (A) tail[43].

### Development of mRNA vaccines

**Figure 1.**
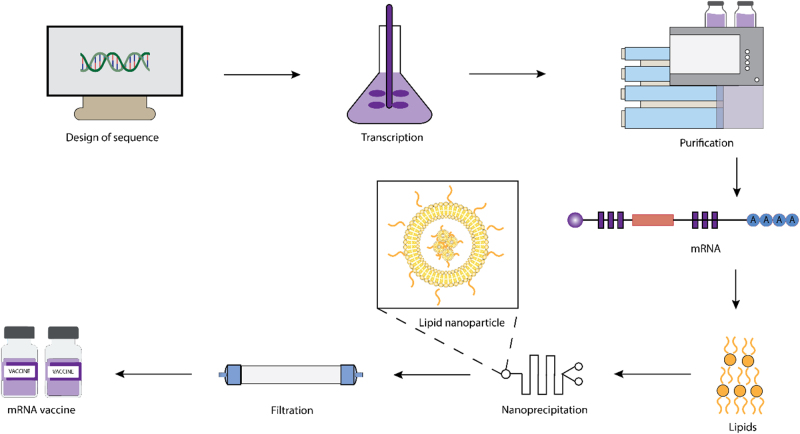
Steps in mRNA vaccine development

To produce an mRNA vaccine against a specific virus, the genome of the virus is first sequenced. After that, a sequence for an antigen of interest is created and inserted into a plasmid DNA construct, which is transcribed into mRNA by bacteriophage polymerases in vitro. The mRNA transcripts are then purified by high-performance liquid chromatography. The purified mRNAs are rapidly mixed with lipids in a microfluid mixer. The process enables lipids to encapsulate mRNAs instantaneously and precipitate as self-assembled nanoparticles. Unencapsulated mRNAs and non-aqueous solvents are removed by filtering or dialyzing the nanoparticle solution. The solution is stored in sterilized vials as mRNA vaccines (Fig. 1)[43].

## Comparative safety and efficacy of mpox mRNA vaccines with traditional vaccines

The rise of mpox as a global health challenge has necessitated the evaluation of various vaccination strategies. While traditional smallpox vaccines have a proven track record, the advent of mRNA vaccines offers novel solutions with distinct advantages. This section critically examines the safety and efficacy of mRNA vaccines for mpox prevention compared to traditional smallpox vaccines.

### Clinical trial data: mRNA vs traditional vaccines

Clinical trials provide the backbone of vaccine development, offering crucial insights into immunogenicity, efficacy, and safety. The emergence of the MPXV as a global health threat has catalyzed the development of vaccines using both traditional and mRNA-based technologies, each with distinct advantages and limitations (Abdelaal *et al*, 2022).

#### Comparative efficacy in preclinical trials

Preclinical studies highlight the superior immunogenicity of mRNA vaccines compared to traditional options. In murine models, Sang *et al*[44] found that mRNA-A-LNP and mRNA-B-LNP vaccines induced a strong MPXV-specific IgG and neutralizing antibodies, surpassing traditional Modified Vaccinia Ankara (MVA)-based vaccines in both humoral and cellular immunity. Similarly, an mRNA-LNP vaccine (mRNA-1769) demonstrated enhanced viral control and reduced disease severity in a lethal MPXV primate model. In this study by Mucker *et al*[45], mRNA-1769 elicited robust neutralizing and functional antibody responses, leading to a marked reduction in viral replication, lesion formation, and disease duration. These findings highlight the potential of mRNA vaccines to offer superior protection against zoonotic orthopoxviruses and mitigate future outbreaks, reinforcing their advantage over conventional vaccine platforms.

While traditional vaccines such as MVA-based vaccines have been instrumental in controlling outbreaks, concerns regarding their durability and scalability persist[46]. The rapid adaptability of mRNA platforms, as demonstrated in SARS-CoV-2 vaccines, suggests their potential for responding to emerging MPXV variants[47]. As preclinical studies progress, comparative efficacy data from clinical trials will be crucial in shaping future vaccination strategies against MPXV and related pathogens.

#### Safety profiles and adverse events

Reactogenicity, or the observable immune response, varies considerably between vaccine platforms. mRNA vaccines, despite inducing robust immune responses, tend to exhibit fewer systemic adverse events compared to traditional live-attenuated vaccines. ACAM2000, for instance, has been associated with a higher incidence of myocarditis, complicating its use in populations with preexisting cardiovascular conditions^[48,49]^. Meanwhile, JYNNEOS demonstrates a more favorable safety profile but requires two doses to achieve comparable efficacy, which can hinder rapid immunization efforts in public health emergencies[13].

Notably, mRNA vaccines are more suitable for immunocompromised populations, a critical consideration given that patients with metastatic malignancies, autoimmune diseases, or those on immunosuppressive therapies are contraindicated for live-attenuated vaccines^[40,48]^. For these groups, mRNA vaccines offer a safer alternative due to their nonreplicating design and lack of live viral components^[50,51]^.

Overall, while both the mRNA and traditional vaccines for mpox have demonstrated strong immunogenicity, the safety profile of mRNA vaccines (like mRNA-1769) appears to be more favorable in the short term compared to traditional live vaccines such as ACAM2000 (Table 1). JYNNEOS, while safer than ACAM2000, requires multiple doses to be most effective and may be less timely in outbreak situations (Deputy *et al*[13]). The primary advantage of mRNA vaccines lies in their rapid development and adaptability, while traditional vaccines benefit from decades of clinical trial data supporting their long-term safety and effectiveness.

**Table 1:** This table highlights the key aspects of short-term and long-term safety profiles between mRNA and traditional vaccines for mpox

Aspect	mRNA vaccines (e.g. mRNA-1769)	Traditional Vaccines (ACAM2000 and JYNNEOS)
Short-term effectiveness	Strong immunogenic responses in early studies with robust neutralizing antibodies and T-cell activation[45]. Reduced symptoms and shorter disease duration compared to traditional vaccines[52].	JYNNEOS provides moderate protection after the first dose but requires a second dose for optimal effectiveness^[13,53]^. ACAM2000 offers immediate immunity but has a higher risk of severe side effects[54].
Short-term safety profile	Lower reactogenicity and fewer side effects compared to ACAM2000[46].	ACAM2000 is associated with high rates of severe side effects, including myocarditis and myopericarditis[48]. JYNNEOS is safer in the short term but requires a longer timeline for full protection^[55,56]^.
Long-term effectiveness	Long-term effectiveness data are still emerging but are promising based on strong early immunogenic responses[57].	Decades of data support the long-term effectiveness of traditional vaccines, especially JYNNEOS, in preventing mpox^[58,59]^.
Long-term safety profile	Long-term safety is being studied, but initial indications suggest fewer risks compared to live attenuated vaccines[52].	JYNNEOS has extensive long-term safety data and is considered relatively safe[60]. ACAM2000 presents long-term safety concerns, especially cardiac complications (Frey *et al*[61]).
Technology and adaptability	mRNA vaccines can be rapidly developed and adapted to address emerging pathogens, as demonstrated during the COVID-19 pandemic[44].	Traditional vaccines, while effective, are less adaptable and slower to produce for new outbreaks[57].

## Future directions and implications

As mRNA technology continues to revolutionize medicine, its potential applications and broader implications demand critical examination. While the success of mRNA-based vaccines during the COVID-19 pandemic highlighted the technology’s immense promise, challenges in scalability, accessibility, and global implementation remain[62]. Additionally, leveraging mRNA technology for future outbreaks and addressing policy gaps will be key to sustaining progress. The following explores the challenges in mass production and administration, the role of mRNA in tackling emerging health crises, and strategic policy recommendations to guide its future development.

### Challenges in mass production, distribution, and administration of mRNA vaccines

**Figure 2.**
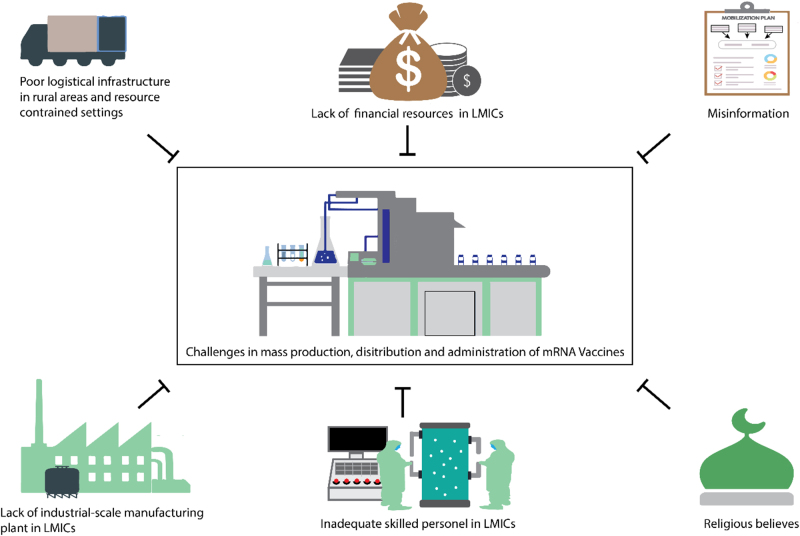
Challenges associated with the production, distribution, and administration of mpox vaccines

The future of healthcare innovation, particularly using novel therapies, vaccines, and technologies, hinges not only on scientific breakthroughs but also on the ability to ensure mass production, distribution, and administration of these advancements. Despite significant progress in research and development, the pathway from a groundbreaking discovery to widespread accessibility remains fraught with challenges (Fig. 2). These issues span various domains, including manufacturing scalability, logistical infrastructure, equitable distribution, and effective administration. Addressing these challenges is vital to achieving the widespread impact envisioned by innovators and stakeholders in the healthcare sector.

One of the foremost challenges in mass production is the transition from small-scale, laboratory-based research to industrial-scale manufacturing[63]. While innovative therapies and vaccines are often developed in highly controlled laboratory environments, scaling up production to meet global demands presents unique difficulties. Many of the latest therapies, such as mRNA-based vaccines or biologics, require intricate manufacturing processes[64]. For example, the production of mRNA vaccines involves advanced biotechnological techniques, including nucleotide synthesis, LNP formulation, and stringent purification processes. These steps demand precision and a controlled environment, which are especially difficult to replicate on a large scale. Any deviation in process parameters can compromise product efficacy and safety, leading to wastage and delays. Establishing facilities capable of producing complex therapies requires significant investment in specialized equipment, cleanroom environments, and skilled personnel. For instance, cell and gene therapies necessitate the use of bioreactors and sterile environments to maintain the integrity of biological materials. Setting up such infrastructure can pose a prohibitive financial burden, particularly for LMICs, exacerbating global inequities in access to cutting-edge medical technologies. The production of medical products often depends on a global network of suppliers for raw materials, reagents, and equipment. Disruptions in this supply chain – as observed during the COVID-19 pandemic – can significantly hinder manufacturing capacity[65]. For example, shortages of essential components like vials, syringes, or LNPs can stall vaccine production, thus delaying immunization campaigns. Scaling up production requires compliance with stringent regulatory standards to ensure product safety and efficacy. This involves conducting additional quality control tests, validating manufacturing processes, and obtaining regulatory approvals for large-scale production. These steps are time-intensive and can delay the availability of medical products, particularly during public health emergencies.

Even if large-scale manufacturing hurdles are overcome, ensuring that medical products reach the intended populations requires a robust logistical infrastructure[66]. The global distribution of medical innovations faces several key challenges. Many vaccines and biologics are temperature-sensitive, requiring storage and transport under controlled conditions to maintain their stability. For instance, the Pfizer-BioNTech COVID-19 vaccine initially required storage at -70°C, a requirement that posed significant challenges for distribution, especially in regions with limited cold chain infrastructure[66]. Developing and maintaining cold-chain systems is particularly challenging in LMICs, where power outages and inadequate refrigeration facilities are common. Distributing medical products across diverse geographic regions often involves navigating physical and political barriers. Remote or rural areas may lack the road networks, airports, or transportation facilities, making it difficult to deliver medical supplies on time. Additionally, geopolitical tensions, trade restrictions, and export bans can disrupt the global flow of medical products, creating disparities in access. The effective distribution of medical products depends on a skilled workforce, including logistics managers, pharmacists, and healthcare workers. Many regions, particularly in LMICs, face workforce shortages, which can impede the distribution and administration of therapies. For example, during the rollout of COVID-19 vaccines, several countries reported challenges in mobilizing enough trained personnel to handle vaccine storage, transport, and delivery[67]. The financial cost of distributing medical products on a global scale is a significant challenge. Expenses related to transportation, storage, and logistics can drive up the overall cost of medical innovations, making them less affordable for resource-constrained settings[67]. Without financial support from governments, international organizations, or private donors, many countries may struggle to bear these costs.

Another critical challenge in mass production and distribution is ensuring equitable access to medical innovations. Despite the emphasis on global health equity, disparities in access persist, driven by several factors. High-income countries often have greater purchasing power and advanced healthcare infrastructure, enabling them to secure a larger share of medical products. In contrast, LMICs may face difficulties in negotiating fair pricing, leading to delayed access to life-saving therapies. For instance, during the initial rollout of COVID-19 vaccines, high-income countries were able to purchase large quantities of doses, while LMICs experienced prolonged shortages[68]. Intellectual property rights and patents can also limit the ability of LMICs to produce generic versions of therapies, even during public health crises. While initiatives like the WHO’s COVAX program aim to address these inequities, challenges remain in balancing the protection of intellectual property with the need for global health equity. Decisions regarding the allocation of limited medical resources often involve complex ethical considerations. Should priority be given to healthcare workers and vulnerable populations, or should distribution be based on geographic need? Addressing these dilemmas requires transparent and inclusive decision-making processes to ensure fairness and public trust.

The final step in the pathway – administering medical innovations to patients – is often referred to as the “last mile.” This step presents unique challenges. First of all, ensuring that patients are aware of and willing to receive new therapies or vaccines is a critical component of successful administration. Misinformation, cultural beliefs, and vaccine hesitancy can hinder uptake, particularly in regions where trust in healthcare systems is low[68]. For example, widespread misinformation about vaccine safety contributed to hesitancy during the COVID-19 pandemic, which delayed efforts to achieve herd immunity. Furthermore, many regions lack the healthcare infrastructure necessary to support the widespread administration of medical innovations. This includes a shortage of healthcare facilities, trained personnel, and essential medical supplies. For example, administering intravenous therapies or complex biologics often requires specialized training and equipment, which may not be readily available in resource-limited settings[69]. The administration of medical products often requires ongoing monitoring and follow-up to assess efficacy and manage potential side effects. For instance, patients receiving gene therapies may need regular checkups to evaluate long-term outcomes. Establishing systems for patient follow-up can be challenging, particularly in regions with weak healthcare systems or limited electronic health record infrastructure. Also, administering medical innovations during public health emergencies requires rapid mobilization of resources and personnel. However, many countries lack the preparedness to respond effectively to emergencies, as evidenced by the chaotic distribution of vaccines and medical supplies during the early stages of the COVID-19 pandemic. Strengthening emergency preparedness is essential to ensure timely and effective administration during crises.

### Role of mRNA technology in future outbreaks

The COVID-19 pandemic has underscored the urgent need for adaptable and rapid medical solutions during global health crises. Among the groundbreaking innovations to emerge from this pandemic, mRNA vaccine technology stands out as a transformative tool with far-reaching implications[70]. Unlike traditional vaccine platforms, mRNA-based approaches allow for the swift design and production of vaccines tailored to specific pathogens. As new infectious diseases continue to emerge – fueled by factors such as climate change, globalization, and zoonotic spillovers – mRNA technology is poised to play a critical role in the future of outbreak preparedness and response. This section explores the potential future applications of mRNA technology, its implications for combating emerging infectious diseases, and its potential to revolutionize public health strategies.

One of the most significant advantages of mRNA technology is its speed. Traditional vaccine development processes often take years, but mRNA platforms can shorten this timeline to weeks or months. For instance, the genetic sequence of SARS-CoV-2 was shared in January 2020, and the first mRNA vaccine candidates entered clinical trials within 2 months[71]. This rapid development timeline is crucial for mitigating the spread of infectious diseases, particularly those with high transmission rates. Scalability is another key advantage. mRNA vaccines can be produced in large quantities using standardized manufacturing processes. Unlike protein-based or inactivated vaccines, which often require complex and time-intensive production methods, mRNA vaccines rely on synthetic processes that are easier to scale. This makes mRNA technology a viable solution for meeting global demand during pandemics. The modular nature of mRNA technology also allows for quick adaptation to new pathogens and variants. Once the genetic sequence of a virus is identified, researchers can design an mRNA vaccine that encodes the relevant antigen. This customizability is particularly valuable for combating rapidly mutating viruses, such as influenza and coronaviruses. This adaptability streamlines vaccine development, enabling mRNA technology to help prevent future outbreaks from escalating into pandemics. Although initial investments in mRNA technology can be high, the long-term cost-effectiveness of this platform is significant. The same manufacturing infrastructure can be used to produce vaccines for multiple diseases, reducing overhead costs. Additionally, the synthetic nature of mRNA vaccines eliminates the need for cultivating live viruses or extracting proteins from biological sources, further lowering production costs[72]. mRNA technology is not limited to vaccine development. It can also be used to produce therapeutic proteins and monoclonal antibodies, offering new avenues for treating infectious diseases[73]. For example, mRNA-based therapeutics could be used to deliver antibodies directly to patients, providing immediate protection during an outbreak. This versatility enhances the overall utility of mRNA platforms in public health.

It is important to note that the success of mRNA vaccines against COVID-19 has sparked interest in applying this technology to other infectious diseases. Diseases such as malaria, HIV, and tuberculosis – which have eluded effective vaccine development for decades – could benefit from the precision and adaptability of mRNA platforms[74]. For instance, researchers are exploring mRNA vaccines that target multiple stages of the malaria parasite’s lifecycle, potentially offering more comprehensive protection. Emerging zoonotic diseases, such as Nipah virus, Hendra virus, and Crimean-Congo hemorrhagic fever, pose significant pandemic threats. mRNA technology offers a promising solution for developing vaccines against these high-priority pathogens. By enabling rapid prototyping and testing, mRNA platforms can accelerate the development of countermeasures for diseases with pandemic potential. Beyond infectious diseases, mRNA technology also holds promise for addressing non-communicable conditions such as cancer and rare genetic disorders[75]. mRNA-based cancer immunotherapies, for example, can be designed to stimulate the immune system to target specific tumor antigens. Similarly, mRNA therapies for genetic disorders can deliver functional copies of defective proteins, offering new treatment options for conditions like cystic fibrosis and muscular dystrophy. While these applications are outside the scope of outbreak response, they highlight the broader potential of mRNA technology to transform medicine.

Despite the remarkable achievements of mRNA technology, global inequities in vaccine distribution remain a significant challenge. Many LMICs lack the infrastructure needed to manufacture and distribute mRNA vaccines, resulting in delayed access during pandemics[67]. Addressing these disparities will require substantial investments in global health infrastructure and technology transfer initiatives. While mRNA vaccines offer many advantages, they also have technical limitations that must be addressed. One major challenge is the requirement for ultra-cold storage and transportation, posing challenges for distribution in resource-limited settings[76]. Advances in LNP formulations and mRNA stabilization techniques are needed to overcome these barriers and ensure broader accessibility. The public perception of mRNA technology can also pose challenges. Vaccine hesitancy, fueled by misinformation and distrust, has hindered the uptake of COVID-19 vaccines in some populations[77]. Efforts to build public trust through transparent communication, community engagement, and education will be crucial for the successful deployment of mRNA vaccines in future outbreaks.

### Policy recommendations and next steps

The path forward requires a multi-pronged approach that addresses manufacturing capacity, global supply chains, healthcare infrastructure, and importantly, ethical and economic considerations. To translate the promise of medical innovations into tangible benefits for all, a series of proactive and collaborative policy actions are necessary. These can be broadly categorized into: (1) investments in infrastructure and supply chains, (2) frameworks promoting equitable access, (3) capacity building and trust, and (4) preparedness and adaptability.

A key imperative is to move beyond centralized manufacturing and establish regional hubs, especially in LMICs. This addresses the overreliance on a few major producers and significantly improves the speed and affordability of access. These hubs should be designed to be versatile and capable of producing a range of medical products, not just those relevant to immediate pandemic responses. The WHO’s mRNA technology transfer hub is a crucial step in this direction, but its success hinges on sustained investment, knowledge sharing, and effective technology transfer. Governments and international organizations should commit to long-term funding and support for the establishment and operation of regional manufacturing hubs in LMICs. This includes providing financial incentives, technical expertise, and logistical support for local production.

Embracing modular manufacturing technologies allows for quicker adaptation to changing needs and improves scalability. Standardized modular units can be assembled and disassembled rapidly, reducing setup times and making it much easier and more cost-effective to scale production up or down as needed. National procurement policies should prioritize modular manufacturing solutions and offer incentives for companies developing and deploying these flexible systems.

Diversifying suppliers, establishing strategic stockpiles of essential components, and leveraging digital technologies for real-time supply chain monitoring are vital for minimizing disruptions. Geopolitical events, natural disasters, and other unforeseen factors can severely impact supply chains; therefore, robust contingency plans and diversified sourcing are critical. To ensure resilience in global health systems, governments must foster greater transparency in supply chains and enforce mandatory disclosure of supply vulnerabilities. International coordination is required to maintain strategic stockpiles of critical inputs and streamline cross-border customs procedures.

The storage and transport of temperature-sensitive products like mRNA vaccines in LMICs presents a significant challenge. Innovation in cold-chain infrastructure is essential, including solar-powered refrigerators, portable cooling solutions, and temperature-stable formulations. Governments should invest in cold-chain infrastructure development, promoting the use of sustainable and energy-efficient technologies. International collaboration is necessary to support the deployment of these advanced cooling systems in resource-limited settings.

Mechanisms such as COVAX and the Medicines Patent Pool are vital to promoting fair access to medical innovations. These initiatives should be strengthened and expanded, moving from a model of donation to a system of shared capacity building. The focus must be on empowering LMICs so that they can manufacture and supply their own needs and those of nearby populations when possible. Increased funding and support for COVAX and similar global health initiatives are also essential to promote equitable access to vaccines and therapies. The scope of the Medicines Patent Pool should be expanded to include a wider range of essential medical products. Intellectual Property (IP) rights must not become a barrier to access during public health crises. While IP protections are crucial for incentivizing innovation, they must not undermine the global good. Policies promoting voluntary licensing agreements and technology transfer facilitate the production of affordable generic versions of therapies in LMICs. This includes the sharing of manufacturing processes and technical expertise, not just raw material.

Incentives from governments can encourage pharmaceutical companies to engage in voluntary licensing agreements and technology transfer, particularly during public health emergencies. International regulations should promote transparency in IP rights and provide mechanisms for compulsory licensing when necessary, with appropriate compensation to innovators. However, this should be the exception, not the rule. Many diseases prevalent in LMICs receive scant attention and resources. mRNA technology presents a cost-effective approach to tackling these neglected diseases, but financial commitment is required to fuel progress. Governments, philanthropic organizations, and global health agencies should prioritize funding for mRNA-based research and vaccine development targeting neglected diseases. Incentive schemes for developing products for less profitable markets are vital.

Healthcare systems must be equipped to administer novel therapies safely and effectively. This encompasses training healthcare professionals, creating telemedicine platforms for remote patient monitoring, and widening access to primary care. Investing in healthcare infrastructure and prioritizing the training of healthcare workers – including vaccine administrators and specialists in advanced therapy delivery – can strengthen preparedness and response capabilities. Telemedicine and digital health solutions should be integrated into existing healthcare systems.

Misinformation and vaccine hesitancy present a considerable obstacle to the successful implementation of medical interventions. Public health campaigns must be culturally sensitive, transparent, and inclusive, leveraging trusted community leaders and digital platforms to disseminate accurate information. A core element must be building trust not only in scientific institutions but also in community and religious leaders. Governments should invest in public health communication initiatives designed to reach diverse populations and counter misinformation. These initiatives should promote science literacy and critical thinking skills and actively engage with community leaders and groups to reach wider audiences and improve engagement.

This ensures resources and expertise can be combined to accelerate the development of solutions to any pressing health threats. Collaborative initiatives like CEPI should be expanded, and more focus must be given to developing solutions for problems that impact LMICs as well as high-income countries. Public–private partnerships and international collaboration in medical research and development should be actively encouraged through joint funding programs and streamlined regulatory processes.

Nations need to prioritize preparedness by creating thorough response plans, conducting regular drills, and establishing teams capable of rapid deployment. International coordination is crucial for any rapid global health crisis. This response must be modular and scalable way using the lessons of both successful and unsuccessful past responses to events and pandemics. Governments should create and regularly update comprehensive national response plans for public health emergencies. International mechanisms for information sharing and coordinated responses should be strengthened through international partnerships. Regular training and simulated exercises should be conducted at every healthcare system level, from the community level to the national government.

Rapid and efficient regulatory pathways are essential for the prompt deployment of novel therapies during any emergency. While preserving robust safety standards, agencies must adopt flexible structures that allow the quick clearance of mRNA-based and other novel medical products. Lessons drawn from the expedited approvals of COVID-19 vaccines should inform future regulatory policy. Regulatory agencies should adopt adaptive frameworks that facilitate fast approvals of novel medical products during public health emergencies. International harmonization of regulatory standards should be a priority, enabling faster access to effective treatments whilst always maintaining high standards of safety.

As mRNA technology advances, ethical considerations surrounding its usage, particularly in relation to genetic modification, increase. Open public discussions are needed alongside clear regulatory frameworks to address concerns and foster responsible implementation. Independent ethical review boards should be established to oversee the development and application of mRNA and other gene-editing technologies. Transparent guidelines should be put in place to ensure that these powerful tools are used ethically and for the benefit of all humankind. Open public dialogue must be a central part of this. The implementation of these policy recommendations will require sustained commitment, collaboration, and resource allocation from governments, international organizations, the private sector, and civil society. The journey from scientific discovery to global health equity is a complex and ongoing effort, but it is an achievable one. By prioritizing these carefully considered steps, we can ensure that the benefits of future medical advances are realized for all of humanity.

## Conclusion

To conclude, the viability of mRNA vaccines as a robust strategy for mpox prevention has been extensively evaluated through this research, contrasting the efficacy, safety, and scalability against conventional smallpox vaccines. Historically, smallpox vaccination has been a landmark success in infectious disease control through its employment of the vaccinia virus; however, since routine immunization has been discontinued after smallpox eradication, immunity has diminished globally, resulting in a dire need for innovation in vaccine technology as mpox outbreaks continue to evolve. mRNA is one such innovation; its rapid design and production capabilities allow for quick interventions in the face of emerging threats and viral mutations. Preclinical findings have shown that mRNA platforms can trigger strong humoral and cellular immune responses that often exceed those seen with MVA-based vaccines. Furthermore, mRNA vaccines with a more favorable safety profile are characterized by lower reactogenicity and decreased incidence of serious adverse effects. The modular and flexible character of mRNA technology also allows modifications to be applied seamlessly, which is essential for addressing the challenges that arise from virus evolution and global pandemics. Nonetheless, there are significant challenges that need to be addressed: scaling up manufacturing processes, quality assurance issues, and establishing efficient cold-chain logistics central for ultra-cold storage requirements. Moreover, equitable global distribution, especially in low-income to medium-income areas, remains a serious policy problem. Through the combination of historical vaccine trends and modern advances in biotechnology, mRNA vaccines represent a paradigm shift in the expansion of global health readiness and resilience against not only mpox but also any infectious disease that may arise in the future.

## Data Availability

No new data was generated.
